# Evaluation of possible effects of crocin against nitrate tolerance and endothelial dysfunction

**DOI:** 10.22038/IJBMS.2019.39604.9389

**Published:** 2020-03

**Authors:** Seyedeh Farzaneh Omidkhoda, BiBi Marjan Razavi, Mohsen Imenshahidi, Maryam Rameshrad, Hossein Hosseinzadeh

**Affiliations:** 1 Department of Pharmacodynamics and Toxicology, School of Pharmacy, Mashhad University of Medical Sciences, Mashhad, Iran; 2 Targeted Drug Delivery Research Center, Pharmaceutical Technology Institute, Mashhad University of Medical Sciences, Mashhad, Iran; 3 Natural Products and Medicinal Plants Research Center, North Khorasan University of Medical Sciences, Bojnurd, Iran; 4 Pharmaceutical Research Center, Pharmaceutical Technology Institute, Mashhad University of Medical Sciences, Mashhad, Iran

**Keywords:** Crocin, Endothelial dysfunction, Malondialdehyde, Nitrate tolerance, Oxidative stress

## Abstract

**Objective(s):**

One of the most important problems of taking nitroglycerin is the nitrate tolerance phenomenon and endothelial dysfunction. Oxidative stress is a high-emphasized one of tolerance mechanisms. The possible effect of crocin, one of the anti-oxidant ingredients of saffron, on the nitrate tolerance model was investigated.

**Materials and Methods:**

In the present study, lipid peroxidation and the level of activated and deactivated forms of eNOS were measured. Animals were administered subcutaneously with 25 mg/kg of nitroglycerin, twice a day for 3 days to induce nitrate tolerance model. For evaluation of crocin effects, 20, 40 and 80 mg/kg/day of this compound were injected intraperitoneally in concomitant with nitroglycerin. In the isolated aorta test, after preparation of aorta rings, different concentrations of acetylcholine, sodium nitroprusside and nitroglycerin were added to the organ bath after inducing contraction by phenylephrine and the responsiveness of tissues was recorded.

**Results:**

Findings showed that nitroglycerin administration caused a remarkable overproduction of malondialdehyde (MDA) in the cells and crocin treatment significantly decreased the MDA level. In the nitrate tolerance group, the level of activated eNOS decreased and the level of deactivated eNOS increased. Crocin partly alleviated these changes: however, its effects were not remarkable. Nitroglycerin injection for 3 days developed tolerance to nitroglycerin and cross-tolerance to acetylcholine (endothelial dysfunction) and sodium nitroprusside. Crocin failed to influence significantly on the nitrate tolerance.

**Conclusion:**

Crocin effectiveness is possibly time-dependent; therefore, increasing the duration of treatment with crocin may lead to a significant prevention of nitrate tolerance and endothelial dysfunction.

## Introduction

Cardiovascular disease (CVD) is one of the most prevalent diseases, which causes approximately 31% of all worldwide deaths every year, based on WHO estimation. 85% of these deaths are the consequence of heart attack and stroke (1). Organic nitrates are common drugs mostly for treating of ischemic heart diseases (IHD) and preventing heart attacks (2). Beneficial effects of nitroglycerin (TNG) on IHD are due to its dilating influence on vessels specially veins, accordingly it causes the reduction in heart preload and afterload, which leads to higher oxygen supply and lower oxygen demand (3). However, there is a challenge with most of these drugs: nitrate tolerance. Continuous taking of nitrates, such as nitroglycerin, for a long time or in high doses, can result in decrease or loss of their efficacy, which is called nitrate tolerance (4). Free drug interval is a method applied for nitroglycerin administration to prevent or at least minimize this tolerance. Based on this method, an interval between two medication intakes is determined in such a way that patient’s body is free of drug for 12 hours, but it has a negative point of high rebound ischemic attacks during this 12 hr interval (5). So, it is necessary to find a way for resolving this problem. 

Nitric oxide (NO), as the cellular active metabolite of nitroglycerin, is produced by mitochondrial aldehyde dehydrogenase 2 (ALDH-2). The dilating effects of NO are exerted through guanylcyclase (GC) activation and increment of cyclic guanosine monophosphate (cGMP) in the cell cytoplasm (6). 

Up to now, scientists have made a lot of efforts to find out the mechanisms involved in the nitrate tolerance phenomenon in order to prevent or reverse it; therefore, several hypotheses now exist, including the reduction of ALDH-2 activity and expression, desensitization of GC activity, hypersensitivity to constriction via higher autocrine release of endothelin and endothelial nitric oxide synthase (eNOS) uncoupling (in this situation eNOS produces free radicals instead of NO), which all of them could be the result of over-generation of free radicals (7). Although, oxidative stress is the most frequent mechanism responsible for tolerance, Beretta *et al.* (2008) revealed that nitroglycerin can partially and irreversibly inhibit ALDH-2 by itself (8). It is also demonstrated that eNOS uncoupling is due to decrease in level of tetrahydrobioptrine (BH_4_), coenzyme of eNOS. The reason of BH_4_ reduction is suppression of GTP-cyclohydrolase I (GCH-I) expression which is involved in BH_4_ production. The eNOS dysfunction can also originate from attenuation of its activating phosphorylation at serine 1177‌ (P‌‌‌‌‌‌‌-eNOS at Ser 1177) or increment of deactivating phosphorylation at threonine 495 (P‌‌‌‌‌‌‌-eNOS at Th‌r 495). In fact, eNOS uncoupling is a molecular mechanism for endothelial dysfunction, which appears during nitrate tolerance (9). 

Crocin, one of the active ingredients of saffron, is prepared from *Crocus sativus *L*. *plant’s stigmas. It is a hydrophilic carotenoid, responsible for the color of saffron. The different traditional therapeutic usages of saffron and its constituents have been shown in various disorders, including inflammation, diabetes, cancer, bronchitis and skin disorders (10, 11). Crocin also possesses significant anti-oxidant effects in different studies and has increased reduced form of glutathione (GSH) level, superoxide dismutase (SOD), and catalase (CAT) expression and decreased malondialdehyde (MDA), NOS, NO and basically the reactive oxygen species (ROS) production (12). The protective effects of crocin have been exhibited against ischemic/reperfusion oxidative injuries in rat skeletal muscles (13), kidney (14), heart (15) and some other organs (16). Crocin has also some specific effects in the vascular system. Imenshahidi *et al.* (2010) investigated the effects of crocin on blood pressure in normotensive and hypertensive rats. Their findings indicated that crocin had hypotensive effect in the dose range of 100-200 mg/kg in normal rats, while in hypertensive rats, this effect was appeared in the lower doses (50-200 mg/kg) (17). Another research revealed that crocin ameliorates endothelial relaxation through ERK and Akt signaling pathways (18).

There are several studies which proposed that applying a natural or a chemical compound with anti-oxidant capacity can prevent or reverse the nitrate tolerance (19-22). Fusi and Sgaragli (2015) successfully used dealcoholized red wine containing polyphenols in an *in* *vitro* model of isolated rat aorta rings to prevent the nitrate tolerance (19). Another study evaluated the protective effects of atorvastatin against tolerance in diabetic and normal rats. The results demonstrated that oral administration of 10 mg/kg/day of atorvastatin for 8 weeks could inhibit tolerance development in both groups of rats (20).

Considering that oxidative stress has been reported as an important reason of the nitrate tolerance and the anti-oxidant effects of crocin have been established in different investigations, the present study was conducted to evaluate the possible preventive effects of crocin against tolerance phenomenon induced by nitroglycerin.

## Materials and Methods

***Materials***

The stigmas of *C. sativus* L. were purchased from Novin Saffron, Ghaen, Iran. DMEM-F12 (Bon Yakhteh, Iran), fetal bovine serum (FBS) (Gibco, USA), trypsin-EDTA (Bon Yakhteh, Iran), phenylephrine HCl (nasal drop 0.5%, Nasophrin ^®^, Sina Darou, Iran), nitroglycerin (ampoules 5 mg/5 ml, 10 mg/2 ml, NITRAL ^®^, CaspianTamin, Iran), sodium nitroprusside (Rottapharm Madaus, Italy), heparin sodium (Darou Pakhsh, Iran), enhanced chemiluminescent (Cat. No.: 32106, Pierce, USA), 30% acrylamide/bis solution (Cat.No.: 61-0156, Bio Rad, USA), poly vinylidene fluoride (PVDF) membrane (Cat. No.:162-0177, Bio Rad, USA), skim milk (Cat. No.: B723, Biomark, India) and tris (Cat. No.: 1.08387, Pars Toos, Iran) were used. Malondialdehyde tetrabutylammonium (Cat. No.: 63287), protease inhibitor cocktail, 2-mercaptoethanol (Cat. No.: M6250), phenyl methan sulfonyl fluoride (PMSF) (Cat. No.: 78830), Tween 20, ethylenediaminetetraacetic acid (EDTA) (Cat. No.: 129K54001V), ethylene glycol tetra acetic acid (EGTA) (Cat. No.: E3889), NaF (Cat. No.: S7920), sodium orthovanadate (Cat. No.: S6508), β-Glycerophosphate (Cat. No.: 50020), sodium desoxycholate (Cat. No.: D6750), penicillin/streptomycin, (4, 5-dimethylthiazol-2-yl)-2,5-diphenyl tetrazolium (MTT), acetyl choline chloride and serotonin HCl were purchased from Sigma-Aldrich, Germany. Thiobarbituric acid (Cat. No.:L-58116980), n-butanol, phosphoric acid, NaCl, KCl, Na_2_HPO_4_, NaH_2_PO_4_, KH_2_PO_4_, CaCl_2_, MgSO_4_, NaHCO_3_, glucose, sodium dodecyl sulfate (SDS) (Cat. No.: 8.22050.1000), N,N,N’,N’-Tetramethyl ethylenediamine (TEMED) (Cat. No.: K39072632), glycin, methanol, ethanol absolute and dimethyl sulfoxide (DMSO) were obtained from Sigma-Aldrich, Germany.

***Crocin preparation***

Crocin was extracted and purified by crystallization method based on the previous study. This method was carried out at different temperatures in the first and second steps, and ethanol 80% was used as the extraction solvent. The purity of crocin in this method is more than 97% (23).

***Animals and cells***

Male Wistar rats were used for this study, with a weight range of 270-300 g. They were kept in the colony room of School of pharmacy, Mashhad University of Medical Sciences, Iran. The conditions were 12/12 hr light/dark cycle at the 21±2 ^º^C temperature and free access to food and water. All animal experiments were conducted in accordance with Ethical Committee Acts of Mashhad University of Medical Sciences. Human umbilical vein endothelial cells (HUVEC) were ordered from Pasteur Institute, Tehran, Iran.

***Cell culture***

Cells (HUVECs) were cultured in DMEM-F12 medium which was modified with 10% (v/v) of FBS and 1% (v/v) of penicillin/streptomycin. The cells were kept in incubator with 37 ^°^C and 5% CO_2_. Experiments were performed in 6 groups in 6 separated 75T flasks. Following treatments were conducted once a day for 3 consecutive days and then MDA and Western blotting test were performed (24).

1. Control: only the culture medium 

2. Nitrate tolerance: 50 μM of TNG solution in the culture medium

3. One μM of crocin in the culture medium

4. Fifty μM of TNG solution and 0.1 μM of crocin in the culture medium

5. Fifty μM of TNG solution and 0.5 μM of crocin in the culture medium

6. Fifty μM of TNG solution and 1 μM of crocin in the culture medium

***MTT test***

To determine the cell viability, MTT test was performed. Firstly, 5×10^3^ cells were seeded in each well of a 96-well plate. After 24 hours, the cells were treated with 0-320 μM of TNG and 0-20 μM of crocin for 72 hours. Culture medium and drugs were refreshed once daily. Then, 10% of MTT stock solution (5 mg/1 ml) was added to the cell medium. After 3 hr, DMSO was added to the wells and the absorbance was recorded in wavelength of 545 nm by ELISA reader (Stat fax-2100, UK).

***MDA test***

The level of cellular lipid peroxidation was measured by MDA byproduct based on thiobarbituric acid reactive substance (TBARS) production. For this purpose, after seeding 1×10^6 ^cells in each flask and performing the above-mentioned treatments, in day 4, the cells lysed with lysing buffer (50 mM Tris-HCl (pH=7.4), 2 mM EDTA, 2mM EGTA, 10 mM NaF, 1mM sodium orthovanadate, 10 mM β-glycerophosphate, 10 mM 2-mercaptoethanol, 0.1-0.2% w/v sodium desoxycholate), protease inhibitor cocktail and phenyl methan sulfonyl fluorid‌‌‌‌e (PMSF) in ice. Cell supernatants were isolated by cold centrifuge in 10000 rpm, for 10 minutes and in 4°C. Total proteins of each sample were measured using Bradford kit. A total of 200 μl of the protein lysates were then mixed with 1.2 ml of 1% phosphoric acid, 0.4 ml of 0.7% thiobarbituric acid solutions and 1.6 ml of n-butanol. The samples were incubated in boiling water for 45 min and after cooling of samples, they were centrifuged at 4000 rpm for 45 min and the absorbance of the isolated upper clear liquid determined at 532 nm. Finally, the amount of MDA was expressed as per mg of total protein (25).

***Western blotting***

For Western blot analysis of P‌‌‌‌‌‌‌-eNOS at Ser 1177, P‌‌‌‌‌‌‌-eNOS at Th‌r 495, AKt and its active form, phosphorylated AKt (P-AKt), in day 4, the cell samples were lysed in lysing buffer ‌‌‌‌‌‌‌‌‌containing 50 mM Tris-HCl (pH=7.4), 2 mM EDTA, 2 mM EGTA, 10 mM NaF, 1 mM sodium orthovanadate (Na_3_VO_4_), 10 mM β-Glycerophosphate, 0.2% W/V sodium deoxycholate, 1 mM ‌‌‌‌‌‌‌PMSF and complete protease inhibitor cocktail. The total proteins were separated on SDS-PAGE gels with appropriate concentration (10%) and transferred to polyvinylidene fluoride (PVDF) membranes. Then, blots were blocked with skim milk (5% during 2 hours for AKt) or with bovine serum albumin (‌‌‌‌‌‌BSA‌‌) (5% during‌‌‌ 2 hours, for P‌‌‌‌‌‌‌-eNOS at Ser 1177, P‌‌‌‌‌‌‌-eNOS at Th‌r 495, P-AKt) at room temperature. After blocking, blots were incubated with 1000-fold diluted primary antibodies‌‌‌: rabbit polyclonal antibody against P-eNOS at Thr495 (Cell Signaling‌‌‌‌‌‌, Cat. No. 9574s), rabbit polyclonal antibody against P-eNOS at Ser1177 (Cell Signaling, Cat. No. 9571s), rabbit monoclonal anti-serum against Akt (Cell signaling, Cat. No. 9272), rabbit monoclonal anti-serum against P-Akt (Cell Signaling, Cat. No. 9271), Mouse monoclonal anti-serum against β-actin (Cell Signaling, ‌‌‌Cat. No. 3700), all for 16-18 hr at 4 ^°^C. Then membranes were washed three times with 0.1% Tween 20 and TBST, and incubated with horseradish-peroxidase conjugated anti-rabbit antibody (Cat. No. 7074 Cell Signaling) or Anti-mouse IgG labeled with horseradish peroxidase (Cell Signaling, Cat. No. 7076) at 1:3000 dilutions for 90 min at room temperature. Finally, protein bands were detected using enhanced chemiluminescence (ECL) reagent and Alliance 4.7 Geldoc (UK) software. Protein bands were analysed using UVtec software (UK). The protein levels were normalized against corresponding β-actin intensities.

***Animal study***

Rats were treated based on following protocols:

1. Control: Intraperitoneal injection of normal saline 0.9% for 3 days

2. N‌‌‌‌‌‌‌‌‌‌‌‌‌itrate tolerance: Subcutaneous‌ injection of 25 mg/kg/dose of nitroglycerin, twice a day for 3 days

3. Crocin: Intraperitoneal injection of 40 mg/kg/day of crocin for 3 days

4. TNG+Crocin 20: Subcutaneous‌ injection of 25 mg/kg/dose of nitroglycerin, twice a day and intraperitoneal injection of 20 mg/kg/day of crocin, twice a day for 3 days

5. TNG+Crocin 40: Subcutaneous‌ injection of 25 mg/kg/dose of nitroglycerin, twice a day and intraperitoneal injection of 40 mg/kg/day of crocin, twice a day for 3 days

6. TNG+Crocin 80: Subcutaneous‌ injection of 25 mg/kg/dose of nitroglycerin, twice a day and intraperitoneal injection of 80 mg/kg/day of crocin, twice a day for 3 days 

***Isolated rat aorta***

In day 4, rats were sacrificed and their aorta rings were isolated, their adhering fats were cleaned and they were cut into rings of 3-4 mm long. All rings were mounted under 2 g resting tension on stainless steel hooks in 20 ml organ baths .These organ chambers were filled with Krebs-Henseleit solution (KHS), with a composition (mM) of: NaCl: 118, KCl: 4.7, MgSO_4_.2 H_2_O: 1.2 KH_2_PO4.2 H_2_O: 1.2, NaHCO_3_: 25, CaCl_2_: 2.5 and glucose: 11.1, and aerated with a mixture of 95% O_2_ and 5% CO_2_ and kept at 37 ^°^C. Tension was measured isometrically by a transducer and a computer-assisted data acquisition system (PowerLab/4SP; ADInstruments, Australia) recorded the changes in isometric tension during the experiments. Aortic rings were pre-contracted with 1 μM phenylephrine and concentration-response curve for TNG (10^-12^-10^-5^), sodium nitroprusside (SNP) (10^-12^-10^-4^ M) and acetylcholine (Ach) (10^-9^-10^-4^ M) were obtained by cumulative addition of these drugs to the bath solution (20, 26). 

***Statistical Analysis***

Results are expressed as mean±SD. Statistical analyses were performed using one-way ANOVA followed by Tukey-Kramer test to compare the differences between means. Differences were considered statistically significant when *P*-value <0.05.

## Results

***Cell viability***

**Figure 1:**
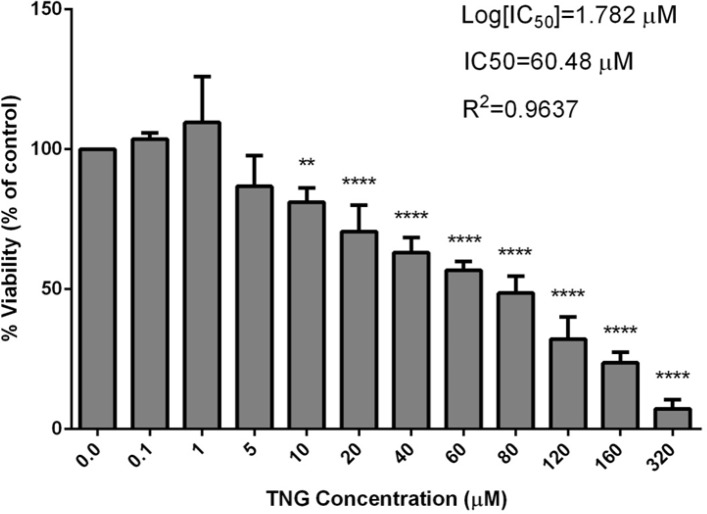
Effect of nitroglycerin on viability of vein endothelial cells

**Figure 2:**
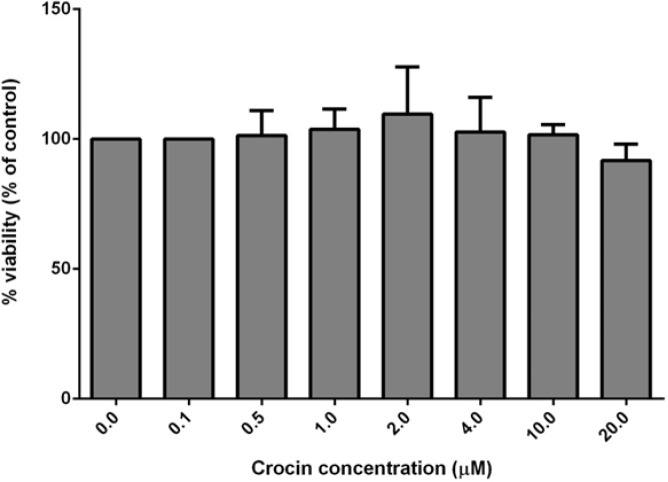
Effect of crocin on viability of vein endothelial cells. The data are displayed as mean±SD (n=4)

Nitroglycerin dose-dependently decreased endothelial cell viability and in the concentration of 60.48 μM, 50% of the cells survived (Figure 1). However, 1-20 μM of crocin did not significantly change cell viability (*P>*0.05) (Figure 2). 

***Lipid peroxidation***

**Figure 3:**
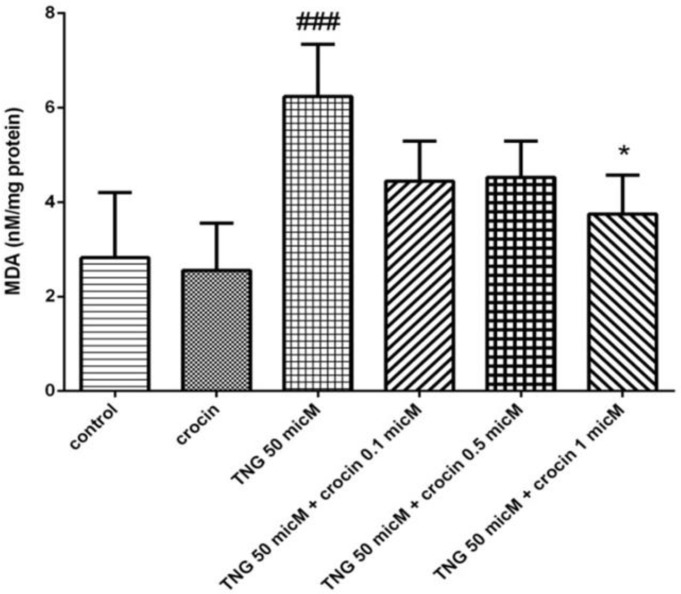
Effect of TNG and crocin treatment on MDA level in vein endothelial cells

In regard to Figure 3, lipid peroxidation which shows the oxidative stress level was markedly increased by 50 μM of nitroglycerin. Crocin with concentration of 1 μM suppressed MDA level significantly (*P<*0.05) (Figure 3).

***The level of activated and deactivated eNOS, Akt and P-eNOS***

**Figure 4:**
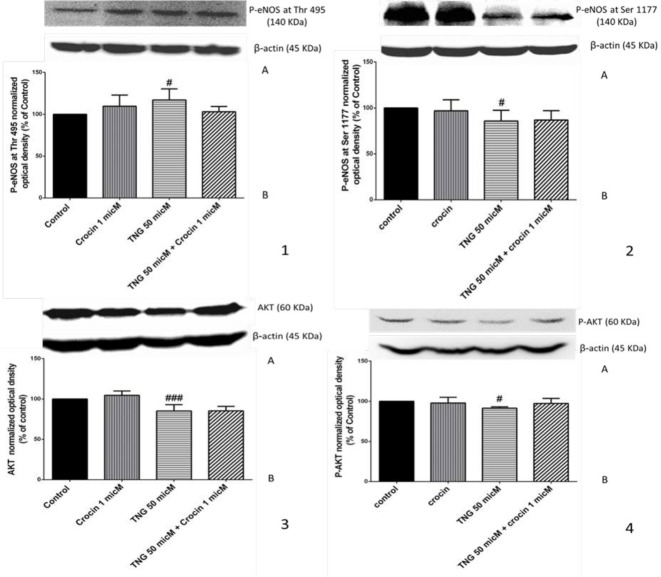
Effect of crocin and TNG treatment on the protein level of P-eNOS at Thr 495, P-eNOS at Ser 1177, Akt and P-Akt

Nitroglycerin increased the level of deactivated eNOS, P-eNOS at Thr 495, and decreased level of its activated form, P-eNOS at Ser 1177. In addition, eNOS phosphorylating (activating) enzyme, Akt and its activated form, P-Akt were significantly suppressed by nitroglycerin. Crocin partially alleviated these changes, but its effects were not remarkable (Figure 4).

***Relaxation responsiveness to TNG***

**Figure 5:**
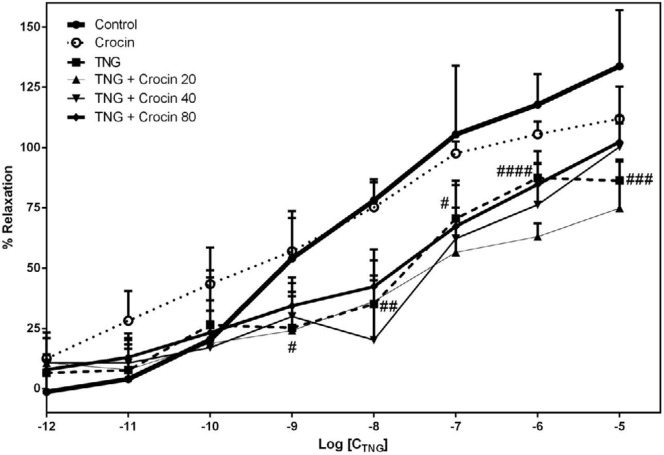
Effect of TNG and crocin treatment on aortic relaxation in response to nitroglycerin. The data are presented as mean±SD (n=6) # *P<*0.05, ## *P<*0.01, ### *P<*0.001, #### *P<*0.0001 vs. control group. TNG: nitroglycerin

Based on data showed in Figure 5, the aortic relaxation response was significantly diminished with 10^-9^-10^-5^ M of nitroglycerin in comparison with the control; however, crocin administration was not able to reverse this effect (Figure 5).

***Relaxation response to Ach***

**Figure 6:**
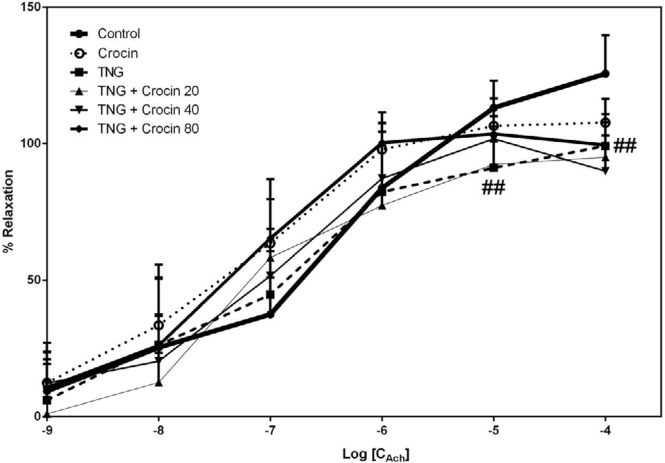
Effect of TNG and crocin treatment on aortic relaxation in response to acetyl choline. The data are presented as mean±SD (n=6). ## *P<*0.01 vs. control group. TNG: nitroglycerin, Ach: acetyl choline

The endothelial responsiveness to acetylcholine was considerably inhibited by nitroglycerin administration in two higher concentrations and there was no significant difference between the crocin plus TNG treated group and the TNG group. In the intermediate concentrations, crocin partially improved endothelial mediated relaxation when compared with control, but it was not statistically significant (Figure 6). 

***Relaxation response to SNP***

**Figure 7:**
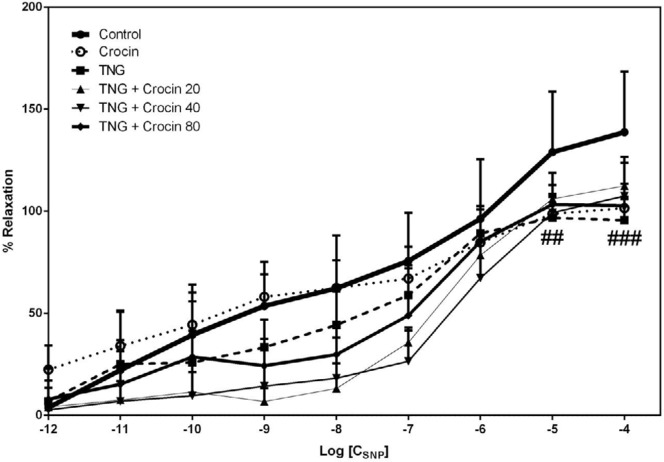
Effect of TNG and crocin treatment on aortic relaxation in response to sodium nitroprusside

In the TNG group, the relaxation percentage in response to SNP was lower than the control group and this difference was significant only at the concentrations of 10^-5^ and 10^-4 ^M. Crocin did not restore the responsiveness of aorta to SNP (*P>*0.05) (Figure 7).

## Discussion

Our findings revealed that nitroglycerin significantly increased lipid peroxidation product which indicates the increased oxidative stress and crocin could markedly inhibit it. The reduction of P-eNOS at Ser 1177, activated form of eNOS, Akt, eNOS activating enzyme and P-Akt as the Akt-activated form were the molecular consequences of the nitrate tolerance in human vein endothelial cells. P-eNOS at Thr 495, as the deactivated form of eNOS was significantly increased in TNG-treated group. Furthermore, the *in vivo* model demonstrated that TNG administration caused decreased relaxation of aorta in response to TNG and endothelial dysfunction which was presented by diminished relaxation response to Ach as an endothelial-dependent vasodilating agent. The results also revealed that TNG administration could lead to a cross-tolerance to SNP, an endothelial-independent NO releasing medicine. In the other hand, 3-day co-treatment of animals with crocin failed to reverse the mentioned influences in spite of its anti-oxidant effect.

Cross-tolerance to acetylcholine and sodium nitroprusside shows that NO-mediated pathway may be disturbed because it is the common pathway between endothelial-dependent and independent NO releasing agents. Some studies also suggested this hypothesis (27, 28).

A similar study was conducted to evaluate the effects of polyphenols in dealcoholized red wine (DRW) on the nitrate tolerance in an *in vitro* model. The Nitrate tolerance was induced by 10 μM of TNG in aorta rings followed by 30 min wash out and then the rings were incubated with DRW (2.8 μg of gallic acid /ml) for 15, 30, 60 and 90 min. Results demonstrated that only the 60 and 90 min-treatment with DRW could significantly reverse the aorta responsiveness to TNG but even not to the normal extent (19). Zhou *et al.* (2017) assessed the effects of Shenmai, a combination of *Ginseng rubra *and *Ophiopogon japonicas, *which was approved by China Food and Drug Administration for heart failure treatment. Unlike our study, they suggested that administration of Shenmai for 14 days (from 7 days before onset of TNG injection) restored the aorta responsiveness to TNG via enhancement of eNOS level, cGMP/cGK-I signaling pathway, enzymatic/non-enzymatic anti-oxidant capacity and reduction of over-generation of free radicals (29). Possibly more extended duration of treatment with protective agents is necessary for prevention of the nitrate tolerance; therefore, our protocol which was 3-day co-treatment of crocin with TNG was not successful to prevent the nitrate tolerance development. There is another similar evidence, for example Imenshahidi *et al. *(2010) exhibited that only 8-week administration of atorvastatin could inhibit the tolerance phenomenon, not 3-day treatment (20), or Tsuneyoshi *et al.* (1989) demonstrated that the injection of 700 mg/kg of N-acetyl cycteine (NAC) in TNG-treated rats could significantly hamper the nitrate tolerance, while the incubation of these rats’ rings with TNG again caused the decreased responsiveness to TNG and then secondly, the incubation of these rings with NAC reversed the tolerance effects. This shows that NAC should continuously administer to have efficacy against the nitrate tolerance (30). In another study, the duration of treatment with serelaxin, a recombinant form of human relaxin-2, was 3 days. This compound activates RXFP1 receptor which causes vessel relaxation via PI_3_K/Akt pathway and it also has anti-oxidant effects. Contrary to our study, serelaxin was successfully used against the nitrate tolerance (21). The treatment duration of serelaxin and crocin was the same but serelaxin had an extra vasodilating mechanism that 20-80 mg/kg/day of crocin did not possess such an effect; however, it is shown that crocin in the higher doses has vasodilating effects which can be via ERK and Akt signaling pathways (17, 18). 

**Figure 8:**
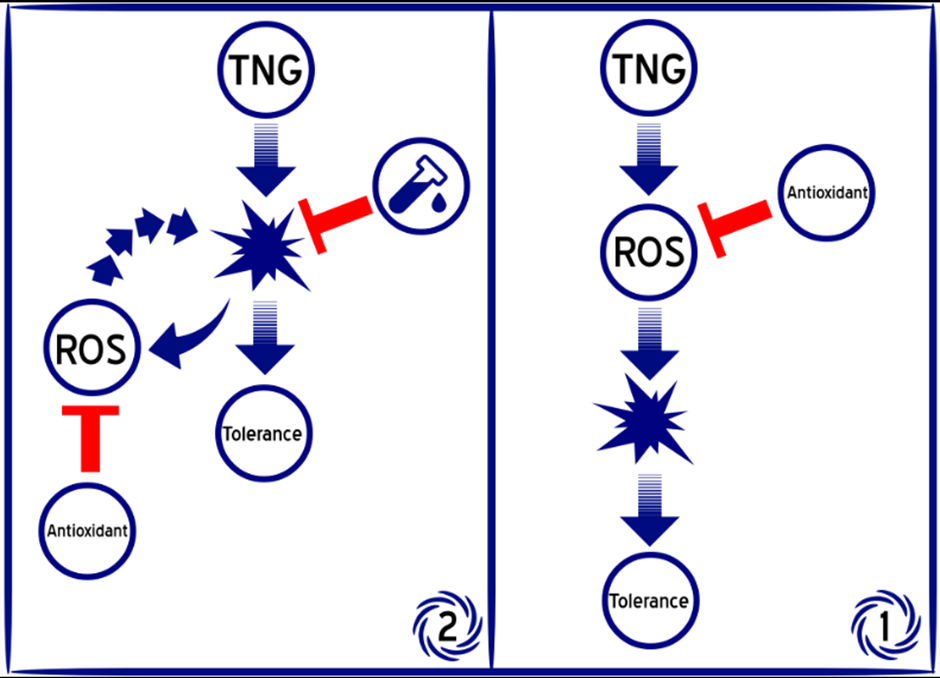
Schematic image of two different hypotheses about the role of oxidative stress in nitrate tolerance development. Part (1): oxidative stress is a primary reason of tolerance, so if a powerful antioxidant agent is used, tolerance can be blocked. Part (2): oxidative stress is the consequence of the original disturbances which can deteriorate it reversely, thus if a powerful anti-oxidant is used, the tolerance blocking is not expected, but it may prevent tolerance exacerbation. In this case, the best way to prevent nitrate tolerance is finding a compound which has the beneficial effects against the main disturbance, in addition of oxidative stress

Considering that the anti-oxidant effects of crocin appeared after 3-day treatment, without any significant effects on the other changes induced by the nitrate tolerance, it can be concluded that possibly oxidative stress is not a crucial and primary reason for tolerance development; however, it can be the consequence of some disturbances induced by TNG and also inversely deteriorates these disturbances. In the other words, oxidative stress is not possibly the primary reason of the nitrate tolerance but it is the result of the other original reasons that some of them were mentioned previously, including partially irreversible inhibition of ALDH-2 which is responsible for bio-activation of TNG and also has the antioxidative effects in the cell (8), or eNOS uncoupling induced by BH_4_ reduction (9). Based on this hypothesis, if an agent which is merely an anti-oxidant is administered, we cannot expect that it completely blocks this tolerance (Figure 8). 

It is reported that pentaerythrityl tetranitrate (PETN), another member of organic nitrates has anti-oxidative effects via increment of heme oxygenase-1 (HO-1) and because of this property there is no tolerance development during its administration, although TNG induces a marked cross-tolerance to PETN, which is much more than other members of organic nitrate (7). A paradox exists in these phenomena, since if oxidative stress is the main mechanism of tolerance, it is concluded that PETN can bypass or compensate the cross-tolerance via its anti-oxidative effects. Therefore, further research is needed.

The data are displayed as mean±SD (n=4). **P<0.01 and **** *P<*0.0001 vs. control group (TNG concentration of 0.0); TNG: nitroglycerin

The data are expressed as mean±SD (n=4). ### *P<*0.001 vs. control group and * *P<*0.05 vs. TNG 50 micM group. TNG: nitroglycerin, MDA: malondialdehyde

(1A), (2A), (3A) and (4A) are Western blot images of P-eNOS at Thr 495, P-eNOS at Ser 1177, Akt and P-Akt. (1B), (2B), (3B) and (4B) were determined using densitometry analysis. The data are presented as Mean±SD (N=4). ###*P<*0.001, #*P<*0.05 vs. control group. TNG: nitroglycerin

The data are presented as mean±SD (n=6). ## *P<*0.01, ### *P<*0.001 vs. control group. TNG: nitroglycerin

## Conclusion

Our findings supported the previous studies on nitrate tolerance mechanisms. Although crocin had anti-oxidant effects, it failed to reverse nitrate tolerance significantly. Because it may have time-dependent effects on this phenomenon and its treatment duration was not possibly long enough to be effective in the present study. The role of oxidative stress in the cellular mechanisms of nitrate tolerance should investigate more accurately.
